# The Carbon Footprint of Biofuels: Can We Shrink It Down to Size in Time?

**DOI:** 10.1289/ehp.116-a246

**Published:** 2008-06

**Authors:** David C. Holzman

Under the Renewable Fuels Standard (RFS), signed into law as part of the Energy Independence and Security Act of 2007, the United States will be producing 36 billion gallons of renewable fuels annually in 2022, with 15 billion of that to come from corn ethanol by 2015. The ethanol conversion plant business is booming. According to the Renewable Fuels Association, a trade group, as of one year ago 114 plants in 19 states had an annual capacity of 5.6 billion gallons per year, and 80 plants—enough to double current capacity—were under construction. All three presidential candidates jumped on the ethanol bandwagon long ago, with Hillary Clinton calling for 60 billion gallons by 2030 at a campaign stop last November (all three have since back-pedaled in the face of concerns about the impact of corn ethanol on food supplies).

The motivation for all this renewable fuel is not just energy independence but also mitigation of global climate disruption, which has been called “the defining challenge of our era” by United Nations secretary-general Ban Kimoon. Among other disquieting scenarios, climate change threatens the world’s food supply. “[T]he current models of climate change impacts on agriculture are showing earlier and more rapid declines in agricultural productivity,” said John P. Holdren, director of the Woods Hole Research Center, in an interview published on the Habitable Planet website. “[T]he models of climate change influence on agriculture do not yet adequately incorporate the effects of a warmer, wetter world on crop pests and pathogens at all. . . . But every ecologist will tell you that crop pests and pathogens do better in a warmer, wetter world.”

Emissions of carbon dioxide (CO_2_), the chief greenhouse gas behind climate change, also threaten to acidify the oceans, decimating the plankton that form the bottom of the oceanic food chain and preventing shell formation in shellfish, according to research described by Elizabeth Kolbert in the 20 November 2006 *New Yorker*. Climate change also threatens to flood most of the world’s major coastal cities.

Transportation accounts for about 27% of anthropogenic emissions of CO_2_, according to the draft *Inventory of the U.S. Greenhouse Gas Emissions and Sinks: 1990–2006,* which the EPA put out for public comment on 7 March 2008. Because they are renewable, biofuels have been held up as probably the fastest and easiest fix to a large part of the carbon problem. Now, two studies published in *Science* on 29 February 2008 have thrown a cold wet blanket on biofuels, claiming that clearing new land and converting existing cropland to produce feedstocks incurs a “carbon debt”—that is, releases more carbon than is saved by the biofuels produced. Moreover, this debt won’t be fully repaid for tens to hundreds of years, depending on the original carbon content of the land, the type of biofuel, and the efficiency of converting the biomass to biofuels. Thus, within the critical time frame for avoiding a climate catastrophe—the next couple of decades, according to the February 2007 United Nations report *Confronting Climate Change: Avoiding the Unmanageable and Managing the Unavoidable*—biofuel crops will only aggravate global climate disruption.

Biofuel proponents argue that the two *Science* papers ignore rising agricultural productivity and the fact that the carbon impact of gasoline—the baseline for comparison—is a moving target, as rising prices encourage exploitation of tar sands and coal liquefaction, which will boost gasoline’s carbon footprint. They assert that several other important factors were also not accounted for: the value of distillers grains, a protein-rich livestock feed that is a by-product of ethanol; rising conversion plant efficiency; and carbon sequestration by alternative agricultural methods such as no-till farming. Some say that creative new ways of practicing agriculture—including some we haven’t yet even imagined—may allow us to have our fuel and eat our food, too. But few question the papers’ theses.

So what is the carbon impact of biofuels? The answer depends upon a slew of unknowns. Will technological advances reduce the production cost of more environmentally friendly biofuels to the point of commercial competitiveness? Can these fuels be grown benignly if market forces offer higher profits when environmental constraints are ignored? Will improvements in yields, conversion efficiencies, and fuel mileage reduce the demand for liquid fuels to the point where biofuels produced on lands not suited for food crops can fuel the world’s demand for transportation? Will civilization show the political will and the regulatory ingenuity to resist the temptation to exploit wild lands for feedstock production when commodity prices spike? Can people change their lifestyles to further reduce demand for farmland? These are the kinds of big questions that will determine whether biofuels help create a sustainable future or an environmental debacle.

## Carbon Debt of the Current Contenders

The world’s soil and plant biomass collectively store about 2.7 times the carbon that is stored in the atmosphere, according to the first *Science* study, whose collaborators included Joseph Fargione, a regional science director with The Nature Conservancy, and David Tilman, an ecology professor at the University of Minnesota. The carbon is sequestered in the form of organic matter that has not fully decomposed—the stuff that gives soil its richness.

The Fargione study showed that on time scales relevant for avoiding severe climate change effects, clearing and plowing virgin land to grow biofuel crops releases more carbon than would be saved by the biofuels made from those crops. According to the report, CO_2_ is rapidly released during burning to clear land or decomposition of leaves and fine roots, then released more slowly at length as larger roots, branches, or wood products decay.

The debt repayment time for biofuels varies from about 17 years for ethanol produced from highly productive sugarcane grown on the Brazilian Cerrado, the world’s most biodiverse savannah, to 420 years for biofuels from palm oil grown in tropical peatland, according to Fargione et al. Converting central U.S. grasslands to corn for ethanol would incur a 93-year note. And growing corn on land that has lain fallow for just 15 years under the U.S. Conservation Reserve Program would require about 48 years to repay the debt.

Moreover, even switching existing croplands from food to biofuel feedstocks would result, albeit indirectly, in similar carbon debts, according to the second article, from a team that included first author Timothy Searchinger, a visiting research scholar at Princeton University. The reason is simple: When land is taken out of food production for any reason, including biofuel production, wild land elsewhere must be plowed to take up the slack in the world’s food supply [for more on this topic, see “Food vs. Fuel: Diversion of Crops Could Cause More Hunger,” p. A254 this issue].

Life-cycle studies that don’t include these indirect effects typically find that ethanol results in a 20% reduction in greenhouse gas emissions relative to gasoline, and biodiesel a 50% reduction. According to the Searchinger article, the authors “found that corn-based ethanol, instead of producing a 20% savings, nearly doubles greenhouse emissions over 30 years and increases greenhouse gases for 167 years.” Moreover, they wrote, projected corn ethanol production in 2016 would use well over one-third of the U.S. corn land harvested for livestock feed in 2004, “requiring big land use changes to replace that grain.” On the other hand, distillers grains could replace a large percentage of the animal feed displaced by fuel crop production.

Besides the direct effect of land substitution, which Searchinger and colleagues determined using a well-established worldwide economic model, there were some more subtle effects that exacerbated the need for replacement farmland. For example, unused land normally sequesters carbon. Thus, any carbon benefit from biofuels crops must exceed the benefit from sequestration if the land is left wild.

Additionally, when U.S. cropland is recruited for fuel crops, the replacement land will likely be elsewhere in the world, where yields typically fall short of America’s science- and technology-enhanced bounty. So an acre of American farmland would need to be replaced by more than an acre of, say, South American grassland or forest, write the authors. And cellulosic ethanol offers little benefit if grown on “American corn fields of average yield,” according to the researchers. The carbon debt: roughly half a century’s worth.

David Morris, vice president of the Institute for Local Self-Reliance in Minneapolis, Minnesota, argues that because corn yields have increased steadily (by 1.6% annually between 1980, when the U.S. corn ethanol program began, and 2006, according to Michael Wang, a vehicle and fuel systems analyst at Argonne National Laboratory), American corn acreage has not grown in a generation, even though ethanol production has skyrocketed from nothing to nearly 5 billion gallons. Morris says 30–50% of the additional corn that will be grown for ethanol under the federal mandate could be met by rising yields without the need for additional land. But Searchinger notes that in a world that is adding 1 billion human beings every 12 years, “every acre of biofuel crops means less land is available to grow food.”

Gasoline’s carbon impact, the baseline for the *Science* study, is itself a moving target. As oil reserves shrink and demand rises, oil companies will turn to harder-to-extract sources, such as Canadian tar sands, and energy-intensive technologies, such as coal liquefaction, all of which will raise that baseline impact. Gasoline from tar sands would increase CO_2_ emissions by 14% over gasoline from petroleum, coal liquefaction by 83%, according to the 2007 Union of Concerned Scientists report *Biofuels: An Important Part of a Low-Carbon Diet*.

Despite criticism from the ethanol industry, plenty of circumstantial evidence supports the concept of second-order land substitution. U.S. biofuel subsidies have encouraged farmers to shift from soybeans to corn, and soybean prices have nearly doubled as production has fallen 19%. At the same time, deforestation and fires have spiked in the main soybean- and beef-producing states in Amazonia, but not in states with little soybean production, wrote William Laurance, a staff scientist with the Smithsonian Tropical Research Institute, in a letter published 14 December 2007 in *Science*. Laurance said these increases are widely attributed to rising soybean and beef prices, with studies suggesting a strong link between Amazonian deforestation and soybean demand.

“Brazil has been making a lot of effort to reduce deforestation, with increased law enforcement,” says Nathanael Greene, a senior energy policy specialist at the Natural Resources Defense Council (NRDC). That effort reduced the rate of deforestation between 2002 and 2004, he says. But then, he notes, grain prices rose, particularly for soybeans, and deforestation in Brazil picked up apace. Indeed, according to an article in the 27 March 2008 issue of *Time*, the rate of deforestation “closely tracks” commodity prices on the Chicago Board of Trade.

The underlying carbon cycle dynamics highlighted in the Fargione an d Searchinger articles are “unequivocally true,” says Greene. He adds, however, that “there are very complicated and important theoretical and modeling questions that still need and deserve a lot of attention before any sweeping conclusions are reached.”

## Cellulosic Biomass: The Feasibility of Futuristic Feedstocks

Cellulosic feedstocks offer the promise of biofuels that are nearly carbon neutral if grown on marginal lands; the feedstocks might even sequester carbon, instead of causing its release from the soil. Moreover, both perennial grasses (including mixed native grasses, miscanthus, and switchgrass) and fast-growing trees (such as hybrid poplars and willows) can be grown with little fertilizer, the fossil fuel–intensive source of about one-third of all direct life cycle carbon emissions associated with agro-industrial corn production. There is also an ample supply of waste cellulose, particularly in the United States. This material, which can include anything from scrap plywood to paper bags, can serve as a carbon-neutral feedstock.

Cellulosic crops also have higher productivity than corn. Perennial grasses sequester large amounts of carbon—and biannual harvesting actually increases sequestration by accelerating the root life cycle, says Tilman. These grasses have large root systems, parts of which are always growing while other parts are dying, adding their ample carbon to the soil. In the course of growing such crops, somewhere between 2 and 5.5 metric tons of CO_2_ will be removed from the air and stored as organic carbon in the soil for each hectare each year, says Tilman.

Yet another advantage of cellulosic biomass that is particularly important given the vast quantities we may end up producing is that they can be grown in polycultures of several different species. This “will be important to reduce the spread of diseases and pests from both an environmental and an economic perspective,” states *Growing Energy: How Biofuels Can Help End America’s Oil Dependence*, a report by the NRDC published in December 2004. “The alternative—increasing application of chemicals—would start to reduce the environmental benefits of switchgrass.”

But it’s still up in the air as to whether cellulosic ethanol will become a commercial reality. It is far more difficult to convert cellulose to ethanol than it is to convert starch. Whereas starch dissolves in water and breaks down into easily fermented sugar molecules, cellulose, although also made of sugars, is insoluble in water.

Complicating matters, cellulose comes intermeshed with hemicellulose and lignin in the woody part of the plant. Hemicellulose is composed of sugar molecules that are harder to ferment than those in cellulose. Lignin, not composed of sugars, cannot be fermented. “You need to chemically pretreat [cellulosic biomass] so enzymes can access the cellulose,” says James McMillan, principal engineer at the National Renewable Energy Laboratory (NREL).

“Consolidated bioprocessing” could greatly reduce the cost of converting cellulose to ethanol, says Lee R. Lynd, a professor of environmental engineering design at Dartmouth College, who published his research on the subject in the February 2008 issue of *Nature Biotechnology*. In such a process, a single microorganism could grow anaerobically on cellulose and ferment the ethanol, eliminating a separate step for enzyme production, he says. This requires no new biological function, merely engineering two existing functions into a single microbe.

A somewhat different approach is being taken by a startup in Warrenville, Illinois. Coskata uses heat to break cellulosic materials down to carbon monoxide, carbon dioxide, and hydrogen, a well-established process. Patented microbes in a proprietary bioreactor then turn that synthesis gas into ethanol. The final step is distillation. One advantage is that there is no need to separate the three components of woody feedstocks. Another is that other carbonaceous materials—even tires—can serve as feedstocks. Chief marketing officer and vice president Wes Bolsen cites a study by Argonne National Laboratory presented in April 2008 at the Fifth Annual World Congress on Biotechnology and Bioprocessing showing that the resulting ethanol reduces greenhouse gas emissions by up to 96% compared with gasoline. A commercial plant is slated to begin operation in 2011, and General Motors has bought a large interest in the company.

McMillan says scale-up remains a big issue for biomass because its energy density—the amount of available energy per unit weight—is low compared with that of fossil fuels: “That makes collecting and transporting it to a conversion facility all the more cumbersome. You can’t just pipe it somewhere, like oil or natural gas.” Additionally, he says, once at the plant, “you have a relatively dilute process, which takes more tanks, more energy for distillation. . . . The capital costs are about double [those of fossil fuels].”

Nonetheless, the NRDC report, which was assembled by a team of mostly academic scientists, including Lynd, stated that “while the logistics of supplying such large volumes of low-density biomass to a single site have not been demonstrated before, systems for doing so can easily be imagined.” The key challenge, he says, is increasing biomass density before transportation, which can be achieved a number of ways—for example, by grinding and compressing the material.

## Algae: Will Scum of the Earth Make Good?

Algae represents another potential feed-stock that is suddenly attracting attention—and venture capital—to dozens of startups. Chevron is working with NREL to produce transportation fuel from algae. The high-risk, high-reward Defense Advanced Research Projects Agency has a project to use it to make jet fuel. Shell built a pilot facility in Hawaii to grow the stuff, which produces an oil that can be converted into biofuel.

Algae has many potential advantages from the point of view of carbon impact. It doesn’t compete with food crops for land or even necessarily fresh water, since many species can grow in brackish or briny water. It reproduces in hours, which means it is potentially far more productive than terrestrial plants. “We might be able to get five thousand gallons [of fuel] per acre per year,” says Al Darzins, a group manager at the National Bioenergy Center at NREL, whereas soybeans only produce about 48 gallons per acre, and corn about 10 times that much. Algae naturally produce oils that have a roughly 50% higher energy content than ethanol and can fit more easily into the current fuel infrastructure. (Conversely, ethanol attracts water, raising corrosion issues in motor vehicles and making pipelines impractical.)

A major part of algae’s productivity potential stems from the fact that a critical limiting factor in plant growth—the very low concentration of CO_2_—can easily be mitigated in water, merely by bubbling in a highly concentrated source, such as the exhaust emissions from a fossil fuel–fired power plant. However, the need for such a carbon source places some limits on locations.

The technology faces a variety of challenges, many of which come down to costs. Algal production systems will need lots of land (sprawling shallow pools give more algae greater access to the sunlight it needs to photosynthesize) and lots of water near a carbon source. Siting will be important, too: Algae can be grown in open waters or in closed systems, but in open shallow ponds in, say, the desert Southwest where there is plenty of sunlight, evaporation and replacement of water would be an issue. Closed systems, on the other hand, would have high capital costs.

Another challenge with algae is that they tend to produce the most oil when starved (this is a way of storing energy when hard times are anticipated), which has negative consequences for reproduction. Algae may ultimately need to be genetically engineered for best results—and plenty of research is going on in this area. But organisms that are engineered for production of a particular product are no longer optimal for survival, and even in closed systems would have to somehow be protected from wild competitors—something Darzins says “nobody knows anything about.”

GreenFuel Technologies of Cambridge, Massachusetts, is one of the more highly regarded of the many startups experimenting with producing biodiesel from algae. The company pipes power plant flue gas into closed systems for a CO_2_ source. Cary Bullock, vice president for business development, says the company’s designs are good for roughly 2,000–7,000 gallons per acre on the basis of a prototype he describes as a kilometer long and about 10 feet wide.

GreenFuel Technologies suffered a setback last summer when the algae in the experimental closed system it was operating with Arizona Public Service reproduced much more quickly than the company had anticipated. The system for harvesting the algae became overwhelmed and had to be shut down as the micron-scale algae clogged the pores of the harvesting membranes. The system also cost twice as much as anticipated. Although the company had to retrench, Bullock casts the event as the normal sort of learning experience that accompanies development of new technologies. Moreover, he sees the unexpectedly high productivity as a portent of the technology’s potential.

Bullock is guarded about details of the systems and plans for the future, saying only that commercialization is still several years away. But *Xconomy*, an online business magazine, reported on 14 March 2008 that the company has signed a $92 million agreement to build an algae-based plant in Europe, contingent on successfully executing a small-scale pilot project.

## The Art of the Conceivable

If biofuels are to play a role in mitigating global warming emissions, there needs to be enough capacity to make significant inroads on swiftly growing petroleum consumption. This is no sure thing. A report published in 2005 by the U.S. Department of Agriculture and Oak Ridge National Laboratory, *Biomass as Feedstock for a Bioenergy and Bioproducts Industry: The Technical Feasibility of a Billion-Ton Annual Supply* (commonly known as the Billion Ton study), determined a biomass potential in the United States of 1.3 billion tons per year, sufficient to replace approximately 30% of current U.S. petroleum consumption (which is expected to roughly double by 2050). The vast majority of this biomass would come from sources that do not compete with food: crop residues, animal manure, residues from construction and demolition debris, and the like, with “relatively modest changes in land use, and agricultural and forestry practices,” according to the report. “This potential, however, should not be thought of as an upper limit.”

Others are more cautious. The report is “generally thought optimistic,” says Jeremy I. Martin, a clean vehicles analyst at the Union of Concerned Scientists, citing the federal RFS’s goal of 21 billion gallons of advanced cellulosic biofuels by 2022 as “a reasonable starting point while we learn more about biomass resource availability.” This is only about one-fifth of the capacity the Billion Ton study describes and 10% of current petroleum use. Martin says a revised Billion Ton study is expected out this summer that will address some of these questions.

But *Growing Energy*, the NRDC’s report, is optimistic about compressing demand to meet potential supply. The report spends several pages describing how to pare the need for land to fuel the nation’s automobiles in 2050 from 1,750 million acres if worked by current practices (compared with the 400 million acres currently farmed) to somewhere between 6 and 48 million acres. The report posits boosting motor vehicle efficiency to more than 50 miles per gallon (easily achievable with today’s technology, as the Toyota Prius demonstrates) to cut gasoline demand from a projected 289 billion gallons in 2050—a figure that accounts for projected 50% population growth—to about 150 billion gallons. The report claims “smart growth” could further reduce demand to 108 billion gallons.

To meet that remaining demand, the report invokes technological advances in conversion efficiency of switchgrass to ethanol, thermochemical conversion of the nonfermentable components of switchgrass to gasoline and diesel, and a boost in field productivity from the current 5 tons per acre per year to about 12.4 tons per acre per year, a reasonably conservative estimate according to various reports. The report also recommends growing switchgrass on much of the 73 million acres currently used to grow soybeans and collecting 75% of the corn stalks and leaves, “stover,” left on cornfields after the grain is harvested (the rest is needed to maintain land quality). Moreover, leaf protein, which constitutes about 10% of switchgrass, could be recovered for animal feed during the production process.

Lynd, a coauthor of *Growing Energy*, predicts a revolution in agriculture as farmers switch from dealing with overproduction, a dominant problem of the last century, to learning “how to get a lot out of a limited land base.” But keeping any revolution sustainable will depend on incorporating agro-industrial carbon cycles—which are only crudely scientifically charted at present—into market incentives and regulatory schemes, despite what Greene calls “the real threat that politics will prove too blunt or too corrupt to make it happen in any optimal way.”

The current federal RFS demonstrates the difficulties in keeping a revolution sustainable. For starters, the standard is not technology neutral; it not only sets goals but also mandates specific means to reach them. Specifically, the RFS calls for biofuels, although renewable electricity or fuels produced with renewable electricity, such as hydrogen, could power cars with less carbon emissions than gasoline.

Then there is a loophole. Although the legislation requires biofuel enterprises to reduce greenhouse emissions by at least 20% relative to gasoline, all conversion facilities that were already being constructed when the bill was signed are exempt. These facilities, which will account for about 80–85% of the 15 billion gallons, could well end up increasing greenhouse emissions—for example, if they are powered by coal.

Greene and Martin prefer California’s Low-Carbon Fuel Standard (LCFS) to the RFS. The California policy, signed into law early in 2007, mandates the reduction of the average carbon life cycle emissions of fuels sold in that state by 10% by 2020 and by 80% by 2050. That forces the oil companies to sell enough biofuel to reduce the average carbon intensity of their fuels overall by the required amount. The standard is technology neutral. Other states, including Washington, Oregon, Arizona, New Mexico, and Minnesota, are considering adopting similar standards.

Although the LCFS is vastly preferable to the RFS, economists tend to prefer cap-and-trade schemes or carbon taxes. Christopher Knittel, an economist at the University of California, Davis, says the LCFS is economically inefficient, because while it essentially taxes gasoline, it subsidizes ethanol. “We don’t want to subsidize ethanol; we just want to tax ethanol with less carbon [than] gasoline at a lower amount than gasoline, because it still generates carbon—just less carbon than gasoline.”

Both carbon taxes and cap-and-trade programs simply tax all fuels—and theoretically any other sources of greenhouse gas emissions—according to carbon content. Thus, they are technology neutral. And the least expensive way to reduce global carbon emissions, economists say, is to tax all emitters equally.

Under a cap-and-trade program, a government sets a limit on emissions and issues permits for only that amount of carbon emission. Polluting companies can either find ways to reduce emissions or buy permits from companies that are better able to reduce their emissions. The Kyoto Protocol, which the United States has not signed, allows its 178 signatories (as of 28 April 2008) to establish cap-and-trade programs with the goal of reducing carbon emissions to 1990 levels by 2012.

## Politics, Politics

Nonetheless, cap-and-trade programs have political vulnerabilities. The European Union’s program has come under heavy criticism because permits were given to the companies rather than auctioned—the European Union therefore essentially subsidizing the permits—and because the permits gave companies credits for more emissions than they were actually using. Because of this, early in 2007 the price of a permit plummeted from a high of US$34 per ton to practically nothing, according to the Worldwatch Institute report *State of the World 2008: Innovations for a Sustainable Economy*.

“The problem with these market-based schemes is that they are inherently vulnerable to corporate lobbying at every stage,” says Kevin Smith, a guest researcher with Carbon Trade Watch in London, a project of the Amsterdam-based Transnational Institute. “Those with the most successful revolving-door relationships received the largest caps.”

Additionally, in what critics feel is a big loophole, companies can buy “offsets,” which *State of the World 2008* characterizes as “cheap credits.” These offsets support projects in developing countries that are intended to reduce emissions. But “[s]uch projects are merely supplementing fossil fuel use; they are not replacing it,” wrote Larry Lohmann, of the British NGO The Corner House, in the Spring 2007 issue of *Synthesis/Regeneration.* “Covering the land with windmills and biofuel plantations will be of little use unless fossil fuel extraction is stopped.”

Cap-and-trade programs don’t necessarily have to be so loophole-laden. For example, the Regional Greenhouse Gas Initiative, a cooperative effort by nine northeastern and mid-Atlantic states, plans to auction permits and is considering using the revenues in part to finance climate-related programs, according to *State of the World 2008*.

Cap-and-trade programs are politically more palatable than anything that has the word “tax” in it, says Knittel. Nonetheless, the Canadian province of British Columbia has instituted a carbon tax. The tax will ultimately rise to US$30 per ton of CO_2_ emitted, or about 30 cents per gallon of gas. The revenue will go back to individuals and businesses in the form of tax cuts elsewhere, says Knittel.

It remains to be seen whether and how cap-and-trade programs, carbon taxes, and other such regulations will address carbon emissions from agricultural land use and land use substitution. A big question is how well policies will work when market incentives prove tempting. If commodity prices rise, farmers will supply more commodity as inexpensively as possible, and one way to do this is to plow new land, says Searchinger. And if regulations constrain that supply, the poor will eat less.

“If ecological solutions are not also economic solutions, they won’t work,” says Greene. “A solution that is not economically sustainable is not environmentally sustainable.”

Still, *Growing Energy* and Greene’s general tenor are optimistic about the possibility of growing low-carbon biofuels, and growing enough of them to portend a reasonably bright future. But a statement in “Biofuels: Not Quite Dead Yet, Thankfully,” an essay posted 8 February 2008 on Greene’s NRDC blog, belies a pessimism about whether human institutions are capable of doing the right thing. “Of course, it is definitely possible (and taking Searchinger’s numbers at face value very likely) that the amount of truly low-carbon biofuels we can drive through real politics and real markets is much smaller than we would hope,” Greene wrote. “This makes the urgency around getting a federal low-carbon fuel standard all the greater.”

## Figures and Tables

**Figure:**
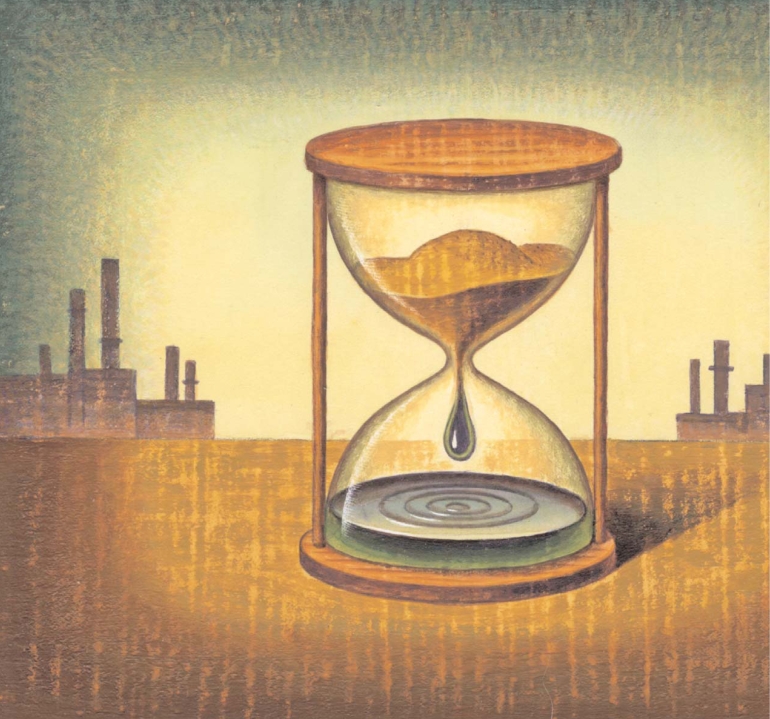


**Figure:**
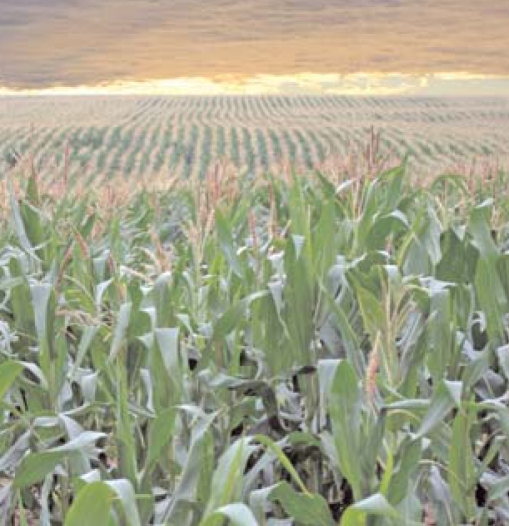
Transportation accounts for about 27% of anthropogenic emissions of CO_2_, the chief greenhouse gas. Biofuels have been held up as a relatively quick and easy fix to a large part of the carbon problem. But two new studies now claim that biofuel production releases more carbon than the fuels themselves save.

**Figure:**
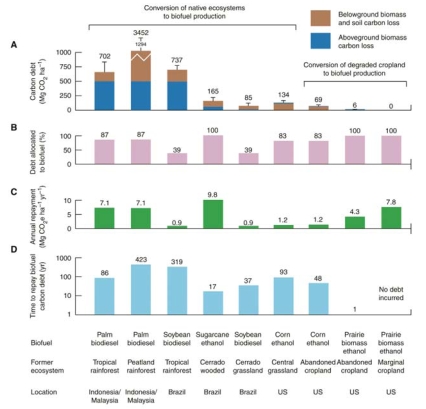
(A) Carbon debt, including CO_2_ emissions from soils and aboveground and below-ground biomass resulting from habitat conversion. (B) Proportion of total carbon debt allocated to biofuel production. (C) Annual life-cycle greenhouse gas reduction from biofuels, including displaced fossil fuels and soil carbon storage. (D) Number of years after conversion to biofuel production required for cumulative biofuel greenhouse gas reductions, relative to the fossil fuels they displace, to repay the biofuel carbon debt. **Source:** From Fargione et al. Science 319:1235–1237 (2008). Reprinted with permission from AAAS.

**Figure:**
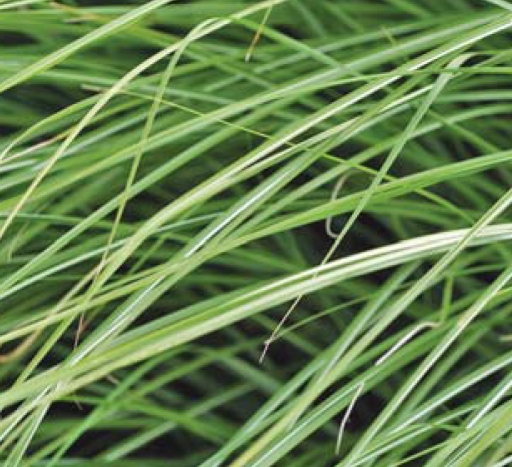
Cellulosic feedstocks offer the promise of biofuels that are nearly carbon neutral if grown on marginal lands; the feedstocks might even sequester carbon, instead of causing its release from the soil. But it’s still up in the air as to whether cellulosic ethanol will become a commercial reality.

**Figure:**
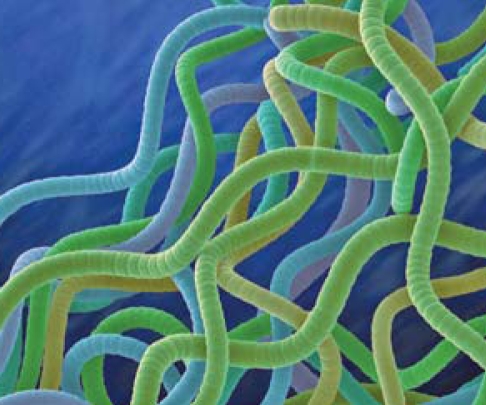
Algae has many potential advantages from the point of view of carbon impact, among them the use of waste CO_2_ to accelerate photosynthesis. The need for a carbon source places some limits on locations.

**Figure:**
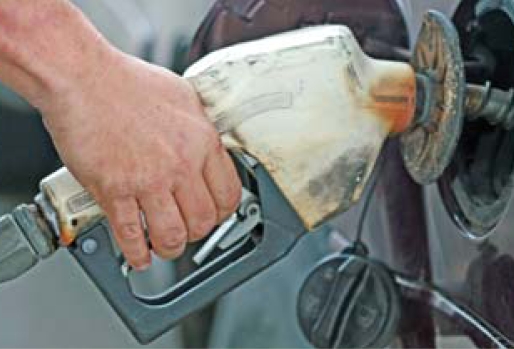
If biofuels are to play a role in mitigating global warming emissions, there needs to be enough capacity to make significant inroads on swiftly growing petroleum consumption.

